# Confirmatory factor analysis of the original posttraumatic growth inventory (PTCI) in a cardiac surgery sample

**DOI:** 10.1186/s41155-026-00395-0

**Published:** 2026-05-09

**Authors:** Débora Grübel Amador, Elisa Kern de Castro, Marcia Moura Schmidt

**Affiliations:** 1 Institute of Cardiology/University Foundation of Cardiology (IC/FUC), Graduate Program in Health Sciences (Cardiology), Princesa Isabel Avenue, 370, Santana, Porto, Alegre, RS 90620-000 Brazil; 2 Psychology Lab, Egas Moniz School of Health and Science, Egas Moniz Center for Interdisciplinary Research, Almada, Portugal

**Keywords:** Posttraumatic Growth, Stress, Psychological Trauma: Coronary Artery Bypass, Factor Analysis

## Abstract

**Objective:**

This study aimed to evaluate the psychometric properties of the Post-Traumatic Growth Inventory (ICPT), a 21-item version adapted to Portuguese.

**Methods:**

The sample consisted of 318 patients who underwent coronary artery bypass graft surgery between October 2022 and March 2024. Participants completed a socio-demographic and clinical questionnaire, the Self-Perceived Stress Scale (EPS-10).

**Results:**

The majority were male (77%), with a mean age of 64.75 years (SD = 9.53), White (93%), and married (70%). Most participants reported experiencing stress (72%) and had an average post-traumatic growth score of 73.9. Confirmatory factor analysis (CFA) supported the original five-factor model of the 21-item scale. Internal consistency estimates ranged from 0.56 to 0.81, with an overall Cronbach’s alpha of 0.90, indicating acceptable reliability. Structural equation modeling further confirmed the factor structure, yielding RMSEA = 0.046, TLI = 0.992, CFI = 0.993, and SRMR = 0.065, demonstrating good model fit.

**Conclusion:**

Overall, the results provide empirical support for the factor structure and reliability of the 21-item version of the ICPT, indicating its suitability for research in the context of chronic diseases.

## Introduction

Post-Traumatic Growth (PTG) refers to the idea that experiencing an adverse situation, such as illness or worsening health conditions, can lead individuals to positively change how they perceive themselves and relate to the world (Tedeschi & Calhoun 1996, 2004). PTG is commonly defined as a positive psychological change that can emerge from the struggle with highly challenging life circumstances, including serious illness or deteriorating health conditions, and encompasses changes in how individuals view themselves and relate to the world (Tedeschi & Calhoun 2004). This process originates from a potentially traumatic or stressful event, often described as a seismic event, which disrupts, threatens, or even dismantles many of the cognitive structures that guide an individual’s understanding, decision-making, and meaning making (Sheikh 2004).

Posttraumatic growth (PTG) encompasses five core domains: greater appreciation of life, more meaningful interpersonal relationships, increased personal strength, the emergence of new possibilities or shifts in life priorities, and enhanced spiritual or existential development (Tedeschi & Calhoun 1996). To operationalize this construct, Tedeschi and Calhoun (1996) developed the 21-item Posttraumatic Growth Inventory (PTGI), which has since been widely used in both clinical and research settings. Following its original development, the PTGI has been translated and culturally adapted into numerous languages, including Japanese (Taku et al., 2007), Spanish (Weiss & Berger 2010), German (Mack et al., 2014; Maercker & Langner 2001), French ( Cadell et al., 2015), Italian (Prati & Pietrantoni 2013), European Portuguese (Teixeira & Pereira 2013), Dutch (Jaarsma et al., 2006) and Chinese (Lau et al., 2015), and is currently available in at least 25 languages (Dubuy et al., 2022).

Across diverse cultural contexts, several studies have employed confirmatory factor analysis (CFA) to examine the factorial validity of the original 21-item PTGI (Jaarsma et al., 2006; Taku et al., 2007; Tedeschi & Calhoun 2004). These CFA-based investigations, conducted in North America, Europe, and Asia, have generally supported the original five-factor structure—Relating to Others, New Possibilities, Personal Strength, Spiritual Change, and Appreciation of Life—although some variability in model specification has been observed across cultural contexts. Overall, CFA results have demonstrated acceptable to good model fit, with certain studies requiring minor model modifications, such as correlated residuals, while retaining the original item structure (Jaarsma et al., 2006; Taku et al., 2007). Reliability estimates derived from these CFA models consistently indicate high internal consistency, with Cronbach’s alpha coefficients for the total PTGI typically exceeding 0.90 and subscale values generally above 0.70 (Jaarsma et al., 2006; Tedeschi & Calhoun 1996). Collectively, these findings support the factorial validity and reliability of the 21-item PTGI, while underscoring the importance of re-examining its measurement structure within specific cultural contexts using CFA.

In Brazil, a validated Brazilian version of the PTGI (PTGI-B) with 18 items was developed by Silva et al. (2016), and its factorial structure was evaluated by Campos and Trentini (2019). These authors concluded that the factor loadings obtained indicated that all 18 items of the PTGI-B appropriately loaded onto their respective factors, resembling the values obtained by Tedeschi and Calhoun (1996). Additionally, an adaptation and factorial validation of the original 21-item version was conducted by Medeiros et al. (2017), which reported internal consistency values ranging from 0.70 to 0.86 and an overall Cronbach’s alpha of 0.92. In these studies, the sample was non-clinical, predominantly female, and composed of individuals with incomplete higher education.

Research has demonstrated that individuals diagnosed with life-threatening illnesses may experience personal growth and positive changes as a result of their struggle with the disease (Purc-Stephenson 2014). PTG has been examined in the literature in relation to various diseases and medical conditions, including cancer (Lelorain et al., 2010), multiple sclerosis (Gil-González et al., 2022), human immunodeficiency virus (Garrido-Hernansaiz et al., 2017), chronic obstructive pulmonary disease (Wang et al., 2021), and coronary artery disease (Waight et al., 2015). Therefore, this study aimed to investigate the factorial structure of the original version of the instrument, as previously adapted by Medeiros et al. (2017) to further expand the understanding of its internal structure at an international level.

Cardiovascular diseases are the leading cause of death worldwide, with coronary artery disease having the greatest impact. According to the World Health Organization, cardiovascular diseases (CVDs) are responsible for more deaths globally than any other cause, accounting for an estimated 19.8 million deaths in 2022, of which the majority are due to heart attack and stroke — conditions closely related to coronary artery disease. These disorders collectively represent a substantial proportion of global mortality and highlight the pervasive burden of CVDs on public health (WHO 2025). Coronary artery bypass grafting (CABG) remains the “gold standard” treatment for patients with multi-vessel coronary artery disease and left main coronary artery stenosis, making it one of the most commonly performed procedures globally (Carrel & Winkler 2017). The procedure restores cardiac blood flow by reconstructing the coronary arteries. It involves the use of either venous or arterial grafts from the patient to bypass the occluded coronary arteries, redirecting blood from the aorta to the coronary circulation. This technique, known as bypass surgery, creates new pathways for myocardial perfusion, allowing the heart to be vascularized by both the pre-existing, affected arteries and the newly grafted bypasses. CABG contributes to the patient’s physical, psychological, and social recovery, significantly improving their quality of life (Martins et al., 2021).

Paradoxically, cardiac surgery is perceived as both a life-saving intervention and a life-threatening event. It is associated with hope for recovery from illness and improved quality of life; however, it is also a significant stressor that can evoke feelings of little or no control over the situation, as well as emotions related to the perceived imminence of death for many patients** (**Rosenberger et al., 2006**)**. Moreover, when patients become aware of their condition and the necessity of surgical intervention, they may experience emotions such as distress, fear, and loneliness, which can increase the risk of traumatic experiences (Tigges-Limmer et al., 2021). While negative symptoms or changes, such as anxiety and stress, are frequently observed, positive psychological transformations may also occur following trauma or major life crises, a phenomenon known as post-traumatic growth (PTG) (Helgeson et al., 2006). This study aimed to confirm the factorial validity and examine the psychometric properties of the original 21-item Posttraumatic Growth Inventory (PTGI) in a sample of patients with chronic heart disease who underwent cardiac surgery, using confirmatory factor analysis. Using the original version allows for direct comparability with previous studies conducted in other chronic disease populations and contributes to a more consistent understanding of posttraumatic growth in this context. It was hypothesized that the original five-factor structure of the 21-item *Posttraumatic Growth Inventory* would be confirmed in a sample of patients with chronic heart disease, with items showing significant factor loadings on their respective factors and overall model fit indices reaching recommended thresholds.

## Methods

### Design

This study is a cross-sectional analysis based on a case series over 17 months, involving patients who underwent coronary artery bypass grafting (CABG) at a cardiology hospital in southern Brazil.

### Participants

Patients aged 18 years or older who underwent CABG and consented to participate in the study were sequentially included. Patients were excluded if they had a tracheostomy or were intubated, had a neurological disorder associated with intellectual impairment, dementia, or were experiencing delirium, as documented in the electronic medical records by the attending medical team. Most of the patients were male (77%), with a mean age of 64.75 ± 9.53 years, 93% were White, and 70% were married. Most patients had an intermediate surgical risk (66.4%). A high prevalence of stress was observed (72%), and the mean post-traumatic growth score was 73.9 points.

### Instruments

#### Perceived Stress Scale (PSS-10)

Stress was assessed using the Perceived Stress Scale (PSS-10). The PSS is a brief scale that measures participants' feelings and thoughts over the past month. The items assess the extent to which respondents perceived their lives as unpredictable, uncontrollable, and overwhelming—three key dimensions repeatedly identified as core components of the stress experience. The scale consists of ten items, including six negatively phrased items (1, 2, 3, 6, 9, 10) and four positively phrased items (4, 5, 7, 8). The response format follows that of the original PSS (Cohen et al., 1983), using a Likert scale ranging from 1 ("never") to 5 ("very often"). To calculate the total score, the four positively phrased items are reverse-scored, and all items are summed, yielding a total score ranging from 0 to 40. Higher scores indicate greater perceived stress (Siqueira et al., 2010).

#### Post-Traumatic Growth Inventory (PTGI)

The PTGI is a 21-item self-report questionnaire that asks participants to rate the extent to which changes have occurred in their lives as a result of a crisis. Responses range from 0 ("I did not experience this change as a result of my crisis") to 5 ("I experienced this change to a very great degree as a result of my crisis"). Scores range from 0 to 105, with higher scores reflecting greater post-traumatic growth. The five PTG factors include New Possibilities, Relating to Others, Personal Strength, Spiritual Change, and Appreciation of Life. Tedeschi and Calhoun (1996) reported a Cronbach’s alpha of 0.92 for the PTGI. In this study, the term "crisis" from the original PTGI was adapted to refer to "cardiac surgery." We used the cross-cultural adaptation of the instrument for Brazilian Portuguese, previously validated in a sample from northeastern Brazil (Medeiros et al., 2017).

### Procedures

Data collection took place between October 2022 and March 2024, during which 318 patients who had undergone coronary artery bypass grafting (CABG) were interviewed. Participants were interviewed, on average, five days post-surgery in the postoperative inpatient unit. During the interview, they provided sociodemographic data and completed the Perceived Stress Scale and the Post-Traumatic Growth Inventory. The PSS-10 was used to assess the surgical experience as a stressful event. The interviewer read the questions aloud and recorded responses for the participants. To complete the PTGI, participants were instructed to reflect on the moment they received the news that they would undergo surgery when responding to the inventory.

### Statistical analysis

The sample size calculation was performed using the PSS Health online version. A sample size of 285 subjects was calculated to detect an effect size (f) of 0.25, chosen by the researcher, to assess the averages of post-traumatic growth by comparing up to three groups (e.g., low, medium and high risco cirurgico), with an increase of 10% for possible losses and refusals; this number should be 318 (Borges et al., 2020). A power of 90% and a significance level of 1% were considered. The factors associated with CPT in this sample are the subject of another article.

The structural validity of the instrument was assessed using Confirmatory Factor Analysis (CFA) to determine whether it retained the same factor structure as the original questionnaire. CFA was conducted using R version 4.3.1 to evaluate the factorial structure of the PTGI. Model fit was assessed using the LAVAAN 0.6–18 package, considering the following fit indices: root mean square error of approximation (RMSEA) with a cutoff point of ≤ 0.06, Tucker-Lewis Index (TLI) > 0.95, comparative fit index (CFI) > 0.95, and standardized root mean square residual (SRMR) with a cutoff point of < 0.08 (Hu & Bentler 1999). Cronbach’s alpha (Reichenheim & Moraes 2007) was used to assess internal consistency and reliability estimation, evaluating the internal consistency of the instrument in Portuguese. Pearson correlation analysis was also performed.

## Results

Between October 2022 and March 2024, 422 patients underwent coronary artery bypass graft surgery at this institution. Of these, 17 declined to participate, 34 died, and 14 were in severe clinical condition, which precluded participation. In addition, 37 patients were discharged before being interviewed, and in two cases the interview was interrupted. Thus, the final sample consisted of 318 patients who completed the interview.

Table 1 presents the mean scores, standard deviations, Cronbach’s alpha values for each domain, and the adjusted Cronbach’s alpha values if specific items were removed. The Cronbach’s alpha values for the five PTGI domains were as follows: Relating to Others (α = 0.81), New Possibilities (α = 0.70), Personal Strength (α = 0.72), Spiritual Change (α = 0.73), Appreciation of Life (α = 0.56), and for the overall scale, α = 0.90. Internal consistency was observed across all five domains.

**Table 1:** Mean, standard deviation, and Cronbach’s alpha for the PTGI

Factors	**Mean**	SD	Cronbach’s Alpha if Item Removed from Total Scale	Cronbach’s Alpha if Item Removed from Domain
Relação com os outros	**3.4**	**1.0**	**0.81**	
06. Tenho uma ideia mais clara de que posso contar com as pessoas em tempos de dificuldade	3.6	1.5	0.90	0.82
08. Sinto-me mais próxima das outras pessoas	3.5	1.5	0.89	0.77
09. Estou mais disponível para demonstrar as minhas emoções	3.4	1.5	0.89	0.78
15. Tenho mais compaixão para com os outros	2.9	1.8	0.89	0.78
16. Dedico-me mais às minhas relações	3.3	1.6	0.89	0.79
20. Aprendi bastante sobre como as pessoas são maravilhosas	3.8	1.2	0.89	0.79
21. Aceito melhor necessitar dos outros	3.6	1.5	0.90	0.79
Novas possibilidades	**3.6**	**0.91**	**0.70**	
03. Desenvolvi novos interesses	3.4	1.5	0.90	0.66
07. Estabeleci um novo rumo para a minha vida	3.7	1.3	0.89	0.63
11.Consigo fazer coisas melhores com a minha vida	3.7	1.2	0.89	0.64
14. Existem outras oportunidades que não teriam existido antes	3.1	1.7	0.89	0.69
17.É mais provável que tente mudar coisas que precisam de mudança	3.8	1.1	0.89	0.65
Mudança pessoal	**3.4**	**1.1**	**0.72**	
04. Sinto que posso contar mais comigo própria	3.1	1.7	0.89	0.66
10.Sei que consigo lidar melhor com as dificuldades	3.1	1.6	0.89	0.60
12. Consigo aceitar o resultado das coisas de forma melhor	3.5	1.3	0.89	0.68
19.Descobri que sou mais forte do que pensava ser	3.8	1.3	0.89	0.68
Mudança Espiritual	**3.0**	**1.6**	**0.73**	
05.Tenho uma melhor compreensão dos assuntos espirituais	2.9	1.8	0.89	0.61
18.Tenho uma maior fé religiosa	3.0	1.8	0.89	0.54
Apreciação da Vida	**3.9**	**0.86**	**0.56**	
01. Mudei as minhas prioridades sobre o que é importante na vida	3.7	1.3	0.90	0.51
02.Tenho uma apreciação maior pelo valor da minha própria vida	4.0	1.1	0.90	0.35
13.Consigo apreciar melhor cada dia	3.8	1.2	0.89	0.52
Total PTGI Score	**3.5**	**0.84**	**0.90**	

Boldface entries indicate the mean values, standard deviations, and Cronbach’s alpha coefficients corresponding to each domain

**Fig. 1:**
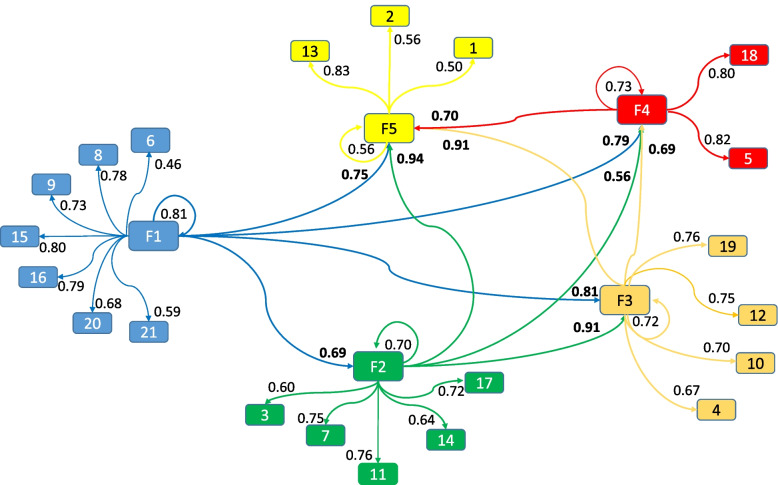
Confirmatory factor analysis – path diagram of the PTGI with standardized factor loadings. F1: Relating to Others, F2: New Possibilities, F3: Personal Strength, F4: Spiritual Change, F5: Appreciation of Life

Confirmatory Factor Analysis (CFA) confirmed the dimensionality and factorial structure of the scale using a structural equation model, which yielded the following fit indices: RMSEA = 0.046, TLI = 0.992, CFI = 0.993, and SRMR = 0.065. Factor loadings ranged from 0.46 (item 6) to 0.83 (item 13), as illustrated in Fig. 1. The factor loadings for each item in the inventory are displayed in a path diagram, where arrows indicate the relationships between variables. Overall, CFA revealed acceptable model fit indices (RMSEA < 0.06, TLI > 0.95, CFI > 0.95, and SRMR < 0.08) (Hu & Bentler 1999).

The confirmatory factor analysis was conducted using the DWLS estimator based on 311 observations. Model fit was evaluated using the scaled chi-square test with a simple second-order correction. The estimated shift parameter was 71.62, indicating substantial noncentrality. Based on the noncentral chi-square distribution (α = 0.05; df = 179), the analysis achieved approximately 95% statistical power to detect plausible deviations from perfect model fit (Moshagen & Bader 2024). Although the total sample comprised 318 participants and no item-level missing data were observed, 311 observations were effectively used in the confirmatory factor analysis due to numerical and estimation requirements of the DWLS estimator based on polychoric correlations.

Table 2 presents the correlations between the five PTGI factors and the total PTGI score. All factors showed strong correlations with the total score: Spiritual Change (*r* = 0.687, *p* < 0.001), Appreciation of Life (*r* = 0.705, *p* < 0.001), New Possibilities (*r* = 0.787, *p* < 0.001), Personal Strength (*r* = 0.825, *p* < 0.001), and the highest correlation, Relating to Others (*r* = 0.844, *p* < 0.001). This indicates that the variance in each of these factors can be explained by the variance in the total score by 47%, 50%, 62%, 68%, and 71%, respectively. Each factor also exhibited significant correlations with the other factors.

**Table 2:** Correlations between PTGI factors

	**Relating to Others**	**New Possibilities**	**Personal Strength**	**Spiritual Change**	**Appreciation of Life**	**PTGI Total**
Relating to Others	1	0.469** < 0.001	0.589** < 0.001	0.534** < 0.001	0.437** < 0.001	0.844** < 0.001
New Possibilities	0.469** < 0.001	1	0.597** < 0.001	0.390** < 0.001	0.610** < 0.001	0.787** < 0.001
Personal Strength	0.589** < 0.001	0.597** < 0.001	1	0.482** < 0.001	0.549** < 0.001	0.825** < 0.001
Spiritual Change	0.534** < 0.001	0.390** < 0.001	0.482** < 0.001	1	0.377** < 0.001	0.687** < 0.001
Appreciation of Life	0.437** < 0.001	0.610** < 0.001	0.549** < 0.001	0.377** < 0.001	1	0.705** < 0.001
PTGI Total	0.844** < 0.001	0.787** < 0.001	0.825** < 0.001	0.687** < 0.001	0.705** < 0.001	1

**p < 0.001 indicates statistically significant correlations at the 0.01 level; *p*

## Discussion

Recent studies have emphasized the importance of considering positive psychological changes following traumatic or highly stressful experiences, underscoring the need for psychometrically sound instruments capable of adequately capturing this process. In this context, the present study aimed to confirm the factorial validity and examine the psychometric properties of the original 21-item Posttraumatic Growth Inventory (PTGI) in a sample of patients with chronic heart disease**.** The results provide robust support for the original five-factor structure proposed by Tedeschi and Calhoun (1996, 2004), confirming that the dimensions of Relating to Others, New Possibilities, Personal Strength, Spiritual Change, and Appreciation of Life are empirically distinguishable and theoretically coherent in this clinical population. The confirmation of the original model is particularly relevant, as it allows direct comparability with previous studies conducted in other chronic illness populations and across different cultural contexts, contributing to a more consistent and cumulative understanding of posttraumatic growth in chronic disease settings. In a comparison of the scores across the five domains of the Post-Traumatic Growth Inventory (PTGI), measured across 21 items in different countries and health conditions (Cheng et al., 2018; de Oliveira et al., 2023; Jeon et al., 2015; Purc-Stephenson 2014), patients in the present study who were in the early postoperative period of CABG exhibited the highest scores, with a mean of 73.95 points.

The Post-Traumatic Growth Inventory (PTGI), originally developed by Tedeschi and Calhoun (1996), was designed to assess positive psychological changes that may emerge following highly challenging life events. Grounded in a theoretical model that conceptualizes posttraumatic growth as a multidimensional construct resulting from the disruption and subsequent reconstruction of core beliefs (Cann et al., 2010), the PTGI has been widely used across diverse traumatic and medical contexts. The Brazilian Portuguese version, validated by Medeiros et al. (2017), retained the original 21-item structure distributed across five dimensions: Relating to Others, New Possibilities, Personal Strength, Spiritual Change, and Appreciation of Life. In line with both the original theoretical framework and subsequent empirical studies, the present findings confirmed the multidimensional structure of the PTGI in patients with chronic heart disease, supporting the assumption that these domains represent distinct yet interrelated aspects of posttraumatic growth. This result is consistent with previous research indicating that the five-factor model of the PTGI remains stable across different cultural contexts and types of adversity, including chronic and life-threatening illnesses (e.g., Taku et al., 2007; Helgeson et al., 2006). Through confirmatory factor analysis, the present study demonstrates that the individual items load meaningfully onto their intended factors, reinforcing the conceptual validity of the PTGI as a theoretically grounded measure of posttraumatic growth rather than a unidimensional index of positive change.

Although Cronbach’s alpha is widely used to assess internal consistency, its interpretation remains a matter of debate, as values above 0.70 are generally considered acceptable, whereas coefficients close to 0.60 may be regarded as adequate in exploratory studies or for research purposes only, but are not recommended for clinical application (Taylor et al., 2003). Moreover, Cronbach’s alpha is sensitive to the number of items comprising a scale; therefore, domains with fewer items tend to yield lower coefficients, potentially leading to an underestimation of internal consistency (Cortina 1993). In the present study, the internal consistency of the five PTGI domains was assessed using Cronbach’s alpha, and all domains demonstrated coefficients above 0.70, with the exception of the Appreciation of Life domain (α = 0.56). This lower coefficient may be explained, at least in part, by the small number of items in this domain, which consists of only three items, one of which was excluded from the Brazilian 18-item version of the PTGI due to inadequate psychometric performance. Given that the primary objective of this study was to verify the theoretically proposed factor structure of the original 21-item version to enable comparisons with international studies involving patients with chronic diseases, the Appreciation of Life domain was retained despite its lower internal consistency. The analysis of Cronbach’s alpha for individual items and the correlations between factors and total PTGI scores demonstrated strong internal consistency. Additionally, removing specific items would have lowered the overall alpha value, indicating that each item is highly correlated with others within its factor and the scale as a whole, further supporting its reliability.

The CFA results indicate that the proposed five-factor structure of the PTGI demonstrates a robust and well-defined latent organization in patients with chronic disease. The excellent fit indices suggest that the observed data closely align with the theoretical model, indicating that the items adequately represent their respective domains of post-traumatic growth. The low RMSEA and SRMR values reflect minimal residual error, while the very high CFI and TLI values indicate that the model explains the covariance structure substantially better than a null model. The consistency of these findings with those reported in the Brazilian validation study (Medeiros et al., 2017) reinforces the structural stability of the instrument across different clinical populations. This convergence provides strong evidence of factorial validity and suggests that the PTGI retains its conceptual integrity when applied to individuals experiencing severe and chronic health-related stressors. Furthermore, the adequate internal consistency observed supports the reliability of the scale for both research and clinical contexts. The use of the original 21-item version enhances cross-cultural and cross-condition comparability, enabling meaningful comparisons of post-traumatic growth across studies, countries, and chronic disease populations. Collectively, these findings support the ICPT as a psychometrically sound instrument for assessing post-traumatic growth in chronic disease settings and underscore its utility for advancing research on psychological adaptation to long-term illness.

Chronic heart disease represents a prolonged and potentially life-threatening condition that can profoundly challenge individuals’ fundamental assumptions about health, control, and mortality (Sawyer et al., 2010; Shand et al., 2015). Unlike discrete traumatic events, chronic illnesses are characterized by ongoing stressors, recurrent medical interventions, and persistent uncertainty, which may repeatedly disrupt core beliefs and necessitate continuous cognitive and emotional adaptation over time (Helgeson et al., 2006). Within this context, posttraumatic growth has been increasingly conceptualized as a relevant psychological process across the trajectory of chronic disease, rather than as a phenomenon restricted to acute trauma exposure. The present findings support the suitability of the PTGI for assessing positive psychological changes in patients with chronic heart disease, as the five dimensions captured by the instrument—such as enhanced appreciation of life, strengthened interpersonal relationships, and perceived personal strength—map onto domains that are especially salient in the experience of living with and recovering from a serious cardiac condition. Importantly, these results are consistent with prior evidence indicating that posttraumatic growth may coexist with ongoing psychological distress and physical limitations in medical populations, rather than implying the absence of suffering (Helgeson et al., 2006).

Among the study limitations, the low internal consistency of the *Appreciation of Life* subscale is noteworthy, compromising its reliability as an autonomous measure. Therefore, its isolated use is not methodologically recommended, and it should be interpreted only within the context of the overall instrument score or as part of the factorial structure of the model. In addition, the sample, drawn from a single hospital and composed predominantly of male participants, may limit the generalizability of the findings to other populations and clinical contexts. Finally, data collection conducted early in the postoperative period (mean of five days) restricts inferences about the temporal development of posttraumatic growth (PTG) and may have introduced social desirability bias, given patients’ tendency to report more positive perceptions in the immediate context of the recovery process.

The present findings are consistent with a growing body of literature demonstrating that posttraumatic growth is a relevant and observable phenomenon in individuals facing chronic and life-threatening medical conditions (Helgeson et al., 2006). The experience of chronic disease may catalyze positive psychological reappraisal and meaning-making processes, even in the presence of ongoing symptoms and functional limitations. Importantly, this body of evidence supports the conceptualization of posttraumatic growth as a dynamic and context-dependent process, influenced by illness severity, treatment stage, and individual coping resources, rather than as a uniform outcome across medical conditions (Tedeschi & Calhoun 2004; Zoellner & Maercker 2006). Within this framework, the confirmation of the PTGI’s factorial validity in patients with chronic heart disease further strengthens its applicability as a standardized measure for capturing growth-related changes across heterogeneous chronic disease populations.

## Conclusion

Patients in the postoperative period following cardiac surgery exhibited levels of posttraumatic growth comparable to those observed in other populations with potentially life-threatening chronic illnesses. In this sample, the PTGI demonstrated robustness in the assessment of positive psychological changes, with high internal consistency (α = 0.90) and preservation of its original five-factor structure. Confirmatory factor analysis corroborated the scale’s dimensional validity, indicating that items are appropriately distributed across the proposed dimensions and that the instrument captures the multidimensional nature of posttraumatic growth in this clinical population. Taken together, these findings demonstrate concordance between the instrument’s empirical organization and the theoretical model underlying its development, supporting its use as a standardized tool for both future research and clinical assessment of growth-related outcomes following cardiac surgery.

## Data Availability

The datasets generated during and/or analyzed during the current study are stored on the Software REDCap® (Research Eletronic Data Capture) and available from the corresponding author upon reasonable request.

## Abbreviations

PTG: Post-Traumatic Growth
PTGI: Post-Traumatic Growth Inventory
PTGI-B: Brazilian version of the PTGI
CABG: Coronary artery bypass grafting
PSS-10: Perceived Stress Scale
CFA: Confirmatory Factor Analysis
